# Fixational eye movements as active sensation for high visual acuity

**DOI:** 10.1073/pnas.2416266122

**Published:** 2025-02-04

**Authors:** Trang-Anh E. Nghiem, Jenny L. Witten, Oscar Dufour, Wolf M. Harmening, Rava Azeredo da Silveira

**Affiliations:** ^a^Département de Physique, Ecole Normale Supérieure, Université Paris Sciences et Lettres, Centre National de la Recherche Scientifique, Sorbonne Université, Université de Paris, Paris 75005, France; ^b^Institute of Molecular and Clinical Ophthalmology Basel, Basel 4031, Switzerland; ^c^Department of Ophthalmology, Rheinische Friedrich-Wilhelms-Universität Bonn, Bonn 53127, Germany; ^d^Faculty of Science, University of Basel, Basel 4056, Switzerland; ^e^Department of Economics, University of Zurich, Zurich 8001, Switzerland

**Keywords:** eye movements, retina, Bayesian inference, adaptive optics

## Abstract

Perception and action are inherently entangled: our world view is shaped by how we explore our environment through complex and variable self-motion. Even when fixating stable stimuli, our eyes undergo small, involuntary movements. Fixational eye movements (FEM) render a stable world jittery on our retinae, which can be expected to harm neural coding. Yet, empirical evidence suggests that FEM help rather than harm human perception of fine detail. Here, we elucidate this paradox by uncovering under which conditions FEM improve or impair retinal coding and human acuity. We combine theory and experiment: model accuracy is directly compared to that of healthy human subjects in a visual acuity task. Acuity is modeled by applying an ideal Bayesian classifier to simulations of retinal spiking activity in the presence of FEM. In addition, empirical FEM are monitored using high-resolution eye-tracking by an adaptive optics scanning laser ophthalmoscope. FEM introduce variability in retinal ganglion cell activity, but they also effectively preprocess inputs to facilitate retinal information encoding. Based on an interplay of these mechanisms, our model predicts a relation between visual acuity, FEM amplitude, and single-trial stimulus size that quantitatively accounts for experimental observations and captures the beneficial effect of FEM. Moreover, we observe that while human subjects’ FEM statistics vary with stimulus size, our model suggests that subjects’ FEM amplitude remains within a near-optimal range, where acuity is enhanced compared to much larger or smaller amplitudes. Overall, our findings indicate that perception benefits from action even at the fine spatiotemporal scale of FEM.

## Significance

Perception is inherently active: we need to move our eyes to see the world around us. Yet our eyes also undergo tiny, unconscious movements that can blur out fine visual details. Paradoxically, previous work suggested that these small movements aid fine detail perception. Here, we investigate this paradox to uncover in which contexts small eye movements help or harm visual acuity. Comparing a model of retinal responses with recordings of human visual acuity, we elucidate the mechanisms by which and conditions in which small eye movements support fine detail discrimination. Our results highlight that perception is active even at the level of very fine eye movements.

Human perception is inherently active: as you read this sentence, your eyes produce incessant, intricate movements necessary for vision. Even when attempting to fixate a single letter without moving, spontaneous, small-amplitude eye jittering occurs, known as fixational eye movements (FEM). Here, we consider FEM generated by ocular drift, whose statistics share similarities with with random diffusion processes, as opposed to microsaccades which are well described by ballistic processes and similar to low-amplitude saccades. In spite of FEM, we perceive our visual environment as stable (1) and we are able to distinguish details finer than the amplitude of FEM (2). Our understanding of the role of FEM in shaping perception remains incomplete: on the one hand, FEM can impair visual acuity by adding uncertainty about stimulus position and shape (2, 3); on the other hand, experiments show that FEM can enhance fine detail vision in human subjects (4, 5).

As evidence suggests that information about the trajectories traced out by the eyes during FEM is unused in downstream processing (3, 6–9), one expects FEM to blur out fine visual details and, thereby, hinder perception. Indeed, retinal spiking is stochastic by nature, and therefore keeping one’s eyes static to overcome noisy spiking is advantageous compared to moving them. From this perspective, a static eye leads to more spikes encoding the same stimulus in the same position, which allows the noise to be averaged out over time. According to this mechanism, FEM can hinder neural coding by preventing noise averaging over time. The influence of FEM on neural coding was modeled in previous studies in which a Bayesian decoder infers stimuli from simulations of stimulus-evoked retinal spiking (2, 3, 10). These studies have demonstrated that stimuli can be successfully decoded from simulated retinal activity in spite of the presence of FEM and have quantified the process. Specifically, pixel-by-pixel stimulus reconstruction accuracy was shown to deteriorate with FEM amplitude (2): vanishing or small FEM allow for a decoder to average out noise, as compared to larger FEM, and it remains possible to decode stimuli in the presence of sufficiently small FEM. More recent work, that considered decoding stimuli made up of combinations of sparse features, indicated that FEM proved useful through a separate mechanism, namely, by moving stimulus features away from gaps in the receptive field mosaic due to heterogeneity in receptive field spacing (10).

In a contrasting picture, human psychophysics suggest that FEM support perception: drift motion was recently shown to be finely tuned to allow subcellular resolution in the fovea when stimuli were produced with adaptive-optics correction, (11). Experimentally suppressing FEM by stabilizing stimuli on a subject’s retina also impairs visual acuity (4, 5). These results emphasize the beneficial role of FEM for fine visual perception. Here, FEM play a helpful role by preprocessing the visual input so as to effectively transform the spatiotemporal structure of receptive fields in the retina (12–14) and thalamus (15, 16). In the temporal domain, FEM renders retinal responses to stimuli less transient in time: while retinal spiking decays over time upon presentation of a static stimulus, jittery eye motion refreshes each cell’s receptive field, which elicits sustained retinal activity that encodes the visual input (12). In the spatial (frequency) domain, the statistics of FEM are such that the preprocessing filter they effectively apply can enhance retinal gain at high spatial frequencies: small-amplitude jitter in eye position causes fine spatial stimulus features to move through retinal receptive fields, which induces temporal changes in receptive field contents and hence stimulus-relevant variations in retinal activity (13, 14). Overall, FEM allow for temporal encoding of stimuli by rendering retinal activity sustained through time and able to encode fine spatial detail through temporal variations in spiking.

Here, we combine theory and experiment to study the role of FEM in shaping retinal coding and visual acuity. In particular, we investigate the interplay of the opposite mechanisms of averaging over noisy spikes, which renders FEM harmful, and temporal coding, which makes FEM advantageous for visual acuity. The two mechanisms are most relevant at different stimulus sizes and FEM amplitudes; we therefore study their interactions and the resulting nature of visual coding across experimental conditions and model parameters.

From the interplay of different coding mechanisms, we derive a relation between visual acuity, FEM amplitude, and stimulus size, which we verify experimentally. In particular, our model accounts for FEM enhancing acuity for stimuli as small as receptive field size or smaller, consistent with previous reports (5, 9). Further, our experimental results show that subjects’ FEM amplitude varies depending on stimulus size. This does not impair retinal coding, and modeling suggests that FEM amplitudes exhibited by subjects remain in a near-optimal range for visual acuity. The results highlight that perception is inherently active even at the level of FEM.

## Results

**Fig. 1.**
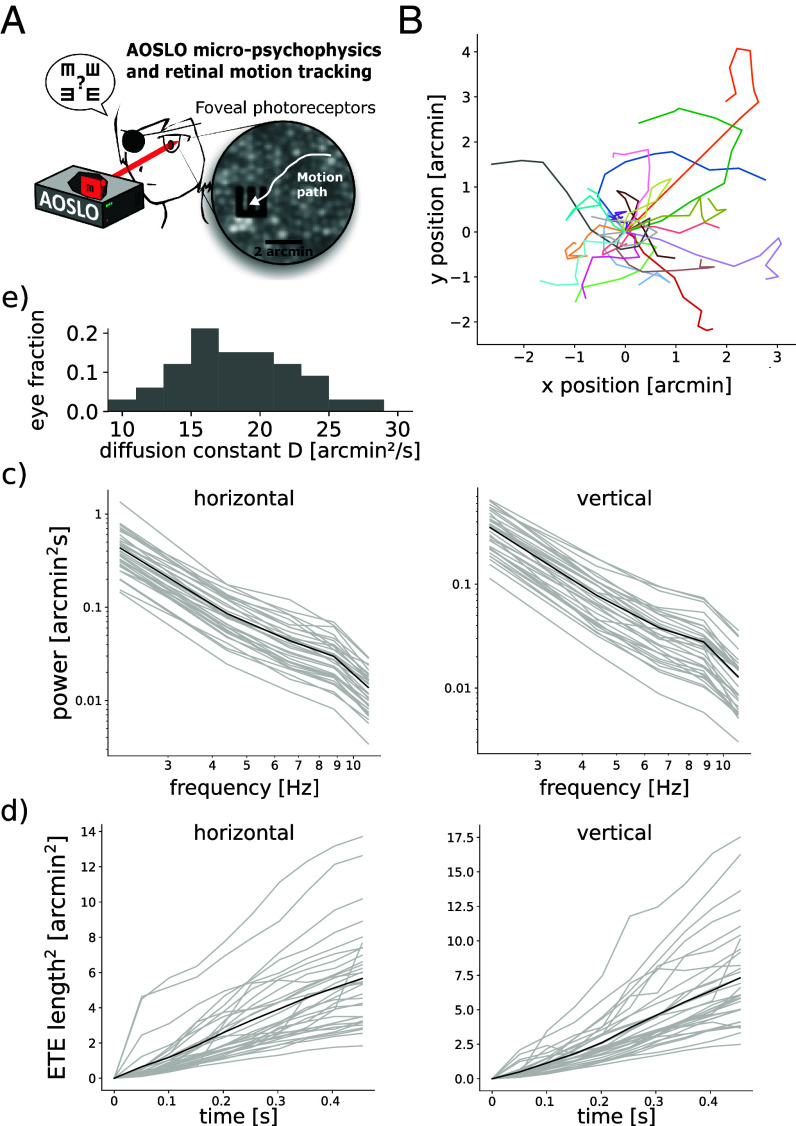
FEM are consistent with a random diffusion process, with diffusion coefficient varying importantly across subjects. (*A*) Experimental set-up and frame captured by the AOSLO, showing a subject’s retinal photoreceptor mosaic with a visual stimulus and recorded FEM trajectory. (*B*) Example FEM trajectories aligned to their starting point for one subject across stimulus presentations (colors). (*C*) Power spectra of FEM trajectories averaged over trials, shown for each subject eye (in gray, mean in black) along horizontal (*Left*) and vertical (*Right*) directions. (*D*) Mean square end-to-end distance of trajectories shown for each subject eye (in gray, mean in black) along horizontal (*Left*) and vertical (*Right*) axes. (*E*) Histogram of diffusion coefficients D, fitted over all trajectories of all trials and stimuli, for each subject. The results report important intersubject variability in terms of D, with the largest D almost three times larger than the smallest value.

To understand how FEM statistics affect visual acuity, we first studied FEM trajectories during a visual discrimination task, in which 17 human subjects had to discriminate between orientations of a Snellen E in a four-alternative forced choice task. The letter E was displayed at multiple sizes, with the thickness of each bar as well as the spacing between adjacent bars of the “E” (Fig. 1*A*) each ranging from 0.3 to 0.8 arcmin. FEM trajectories were recorded through eye tracking with an adaptive optics scanning laser ophthalmoscope (AOSLO) (one subject’s example trajectories shown in Fig. 1*B*). The results revealed trajectories consistent with a random diffusion process, in that power scales as $1/f^{2}$ with frequency f (Fig. 1*C*), as expected from the literature (17), and the squared end-to-end length of trajectories was shown to grow linearly with time (Fig. 1*D*; see *SI Appendix* for derivations in the case of a diffusion process). To quantify the proportionality coefficient of this relation, we fit the diffusion coefficient D, computed as the slope of the two-dimensional mean square displacement across a time step of 50 ms and has the dimension of ${\mathrm{arcmin}}^{2}/s$ (see *Materials and Methods* for details). We find that D varies substantially across subjects (Fig. 1*E*).

**Fig. 2.**
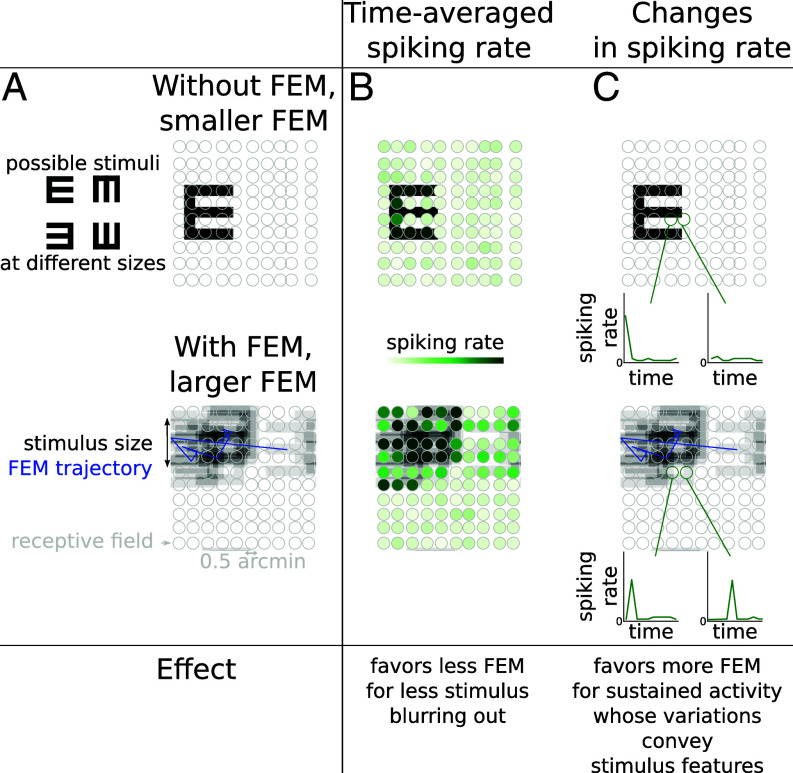
Mechanisms by which FEM affects retinal responses and stimulus encoding. (*A*) Snellen E stimulus (black) moving alongside schematic FEM trajectory (blue) and projected onto RGC receptive fields (gray) for stable stimuli (*Top*) and jittery stimuli in the presence of FEM (*Bottom*). Each letter E represents the input to the retina at a different point in time. (*B*) Time-averaged spiking rate (green) elicited for each RGC by moving stimuli. In this coding scheme, large FEM result in a blurred-out stimulus. Conversely, less to no FEM are favored as they allow to average out noise from stochastic spiking activity by keeping the eye relatively stable through time. (*C*) Changes in the spiking rate of two example RGCs due to variations in receptive field content caused by FEM. In the absence of FEM, light intensity is constant through time in each receptive field, leading to RGC activity decaying in time. In the presence of FEM, however, RGC responses to stimuli are sustained, and changes in spiking rate can convey stimulus-relevant information.

How do FEM affect visual discrimination? From a theoretical point of view, one must consider the interplay between two opposing coding mechanisms which involve the statistics of FEM, controlled by D, the stimulus properties, and the response properties of retinal cells (Fig. 2*A*). On the one hand, coding benefits from averaging over noisy spikes by keeping the eyes stable (2). Averaging over noise especially supports acuity in the case of coarse stimuli, where stimulus features are as wide as receptive fields or wider, and stimulus orientation can still be identified when the stimulus is downsampled into the space of retinal receptive fields (Fig. 2*B*).

On the other hand, temporal encoding of visual stimuli by the dynamics of retinal activity benefits from FEM. Indeed, FEM refresh the content of receptive fields, which leads to more sustained stimulus encoding over time, as well as allow for FEM-induced temporal fluctuations in retinal responses, which can convey information about small stimulus details. Indeed, FEM permit multiple “samples” of a same stimulus to be presented at different retinal positions over time, causing a larger number of retinal ganglion cells (RGCs) to be involved in the encoding of a stimulus and, in particular, of its fine spatial details. We note that using motion to present stimuli at different positions is a strategy also used to enhance resolution in astronomy and in some smartphone cameras (18). For example, in Fig. 2*C* (*Bottom* row), the stimulus moves in and out of a given receptive field under the effect of FEM, which results in changes in retinal spiking rate that encode relevant information about stimulus position and orientation. Temporal coding rendering FEM advantageous is consistent with FEM effectively transforming the structure of spatiotemporal receptive fields (12, 13), which is believed to enhance retinal sensitivity to fine stimulus details (5). This mechanism appears particularly relevant for stimulus features finer than receptive field size, for which retinal encoding amounts to downsampling, and intermediate FEM amplitudes. For small or vanishing FEM, receptive field contents are constant and retinal activity therefore decays over time (Fig. 2*C*, *Top*). Conversely, for FEM larger than the size of the small details, the content of the receptive field of each cell varies dramatically from one region of the stimulus to a different region to which it is moved by FEM; this prevents any averaging over noisy RGC spiking and, hence, harms acuity. From the interplay between these coding mechanisms, one expects that visual acuity depends nontrivially on both stimulus size and FEM amplitude.

To explore the combined effect of these mechanisms, we investigated a simple model of retinal responses in the presence of FEM. As in previous simulation works (2, 3, 10), we modeled FEM as resulting from a two-dimensional diffusion process with step size drawn from a Poisson process, controlled by a diffusion coefficient D (see *Materials and Methods* for details). Indeed, empirical evidence supports that, in the specific context of our stimuli and task unlike in other contexts (see, e.g., ref. 19), diffusion is isotropic (*SI Appendix*, Fig. S1 *A* and *B*); it can thus be modeled by the same coefficient, D, over both horizontal and vertical axes. Furthermore, while temporal correlations exist in the empirical FEM trajectories, these are limited to short time scales (*SI Appendix*, Fig. S1 *C* and *D*). For time scales longer than 50 ms, which coincides with the time scale of the kernel of temporal receptive fields, the empirical diffusion process can be well-approximated by an independent diffusion process.

The spiking activity of RGCs was modeled at the foveola, assuming one-to-one correspondence with photoreceptors (20), in response to stimuli moving in the retina’s reference frame. RGC receptive fields were arranged into a mosaic (Fig. 1*A*). Each RGC’s spiking rate in response to stimuli was characterized by a spatiotemporal kernel: spatial receptive fields were Gaussian with circular symmetry, and temporal receptive fields rendered RGCs sensitive to temporal changes in light intensity, as in transient RGCs. From simulated spiking rates, RGC spikes were drawn according to a Poisson process. The only free parameters were the temporal kernel time scale as well as the gain of RGCs and baseline level of RGC activity, which controlled the sensitivity to stimuli and the spiking rate in the absence of stimuli, respectively. The parameters were set based on ex vivo recordings in the primate fovea (21). To scale the firing rate to match the light contrast in our experiments, we used measurements of the firing rate dependence on contrast from in vivo neurons which receive direct input from RGCs. Hence, it was possible to estimate the likelihood of spiking given the stimulus precisely in the model.

Stimulus-relevant evidence conveyed by RGC spikes was extracted from spikes in a Bayesian inference approach, similar to a Kalman filter, as the time evolution of a posterior distribution of both the stimulus orientation and its position. A Bayesian classifier was used as an ideal model for how the rest of the brain processes information from retinal spikes, following past work (2, 3, 10). This classifier was ideal in that it made optimal use of the posterior: all the stimulus-relevant information available in spiking activity was employed to yield the best possible accuracy at classifying stimuli, given the simulated retinal spikes. The classifier had access to the form of the likelihood of spiking given the stimulus, as well as to the statistics of the eye movements, i.e., D, but not the specific trajectories. In both past implementations of inference (2, 3, 10) and in our case, an important subtlety is that the position of the stimulus on the retina has to be inferred from spikes, in the absence of a motor efference copy providing information about the oculomotor commands giving rise to FEM. While past work (2, 3, 10) considered the inference of the full image, our model classified only the orientation of the image. This simpler setting was chosen to make the theoretical results amenable to a comparison with human behavior in our psychophysical experiments.

Starting with no prior knowledge about the stimulus and hence a flat prior distribution, evidence was accumulated from RGC spiking patterns, and the posterior distribution was updated as follows:

[1] $$\begin{array}{cc} \underset{\mathrm{posterior}\;\mathrm{distribution}}{\underbrace{P(\lambda ,x,y|{\left\{\sigma_{t^{\prime }}\right\}}_{t^{\prime }\leq t})}} & =\frac{1}{Z_{t}}\sum_{x^{\prime }}\sum_{y^{\prime }}\underset{\mathrm{likelihood}}{\underbrace{P(\sigma_{t}|\lambda ,x,y,x^{\prime },y^{\prime })}}\\ & \;\times \underset{\mathrm{transition}\;\mathrm{matrix}}{\underbrace{P(x,y|x^{\prime },y^{\prime })}}\underset{\mathrm{prior}\;\mathrm{distribution}}{\underbrace{P(\lambda ,x^{\prime },y^{\prime }|{\left\{\sigma_{t^{\prime }}\right\}}_{t^{\prime }\leq t-1})}}, \end{array}$$

where λ denotes the stimulus orientation (top, bottom, left, or right), x and y are its center position in the retina reference frame, ${\left\{\sigma_{t^{\prime }}\right\}}_{t^{\prime }\leq t}$ is the history of spiking patterns across RGCs up to time t, and $Z_{t}$ is a normalization constant. $P(\sigma_{t}|\lambda ,x,y,x^{\prime },y^{\prime })$ is a product of $P(\sigma_{t}^{i}|\lambda ,x,y,x^{\prime },y^{\prime })$ over all RGCs i (*SI Appendix*) since the cells are independent and any correlation between their activities comes from the stimulus. $P(x,y|x^{\prime },y^{\prime })$ is the transition matrix governing the diffusion process of FEM, characterized by the diffusion coefficient D (*SI Appendix*); namely, the probability that the stimulus moves, during one time step, from coordinates $\left(x^{\prime },y^{\prime }\right)$ to coordinates (x,y). We note that the likelihood’s dependence on $\left(x^{\prime },y^{\prime }\right)$ is due to the presence of a temporal kernel in our model. As a result, the probability of a spike pattern depends on both the present and previous stimulus positions.

The orientation of the E for which the posterior was maximized is the classifier’s outcome: $\lambda^{*}=\;{\text{argmax}}_{\lambda }\Sigma_{x}\Sigma_{y}P\left({\left\{\sigma_{t^{\prime }}\right\}}_{t^{\prime }\leq t}|\lambda ,x,y\right)$. Repeating this procedure for 50 trials, the fraction of correct discrimination was estimated after 500 ms of stimulus presentation (to match experimental conditions), for each value of D and each stimulus size.

**Fig. 3.**
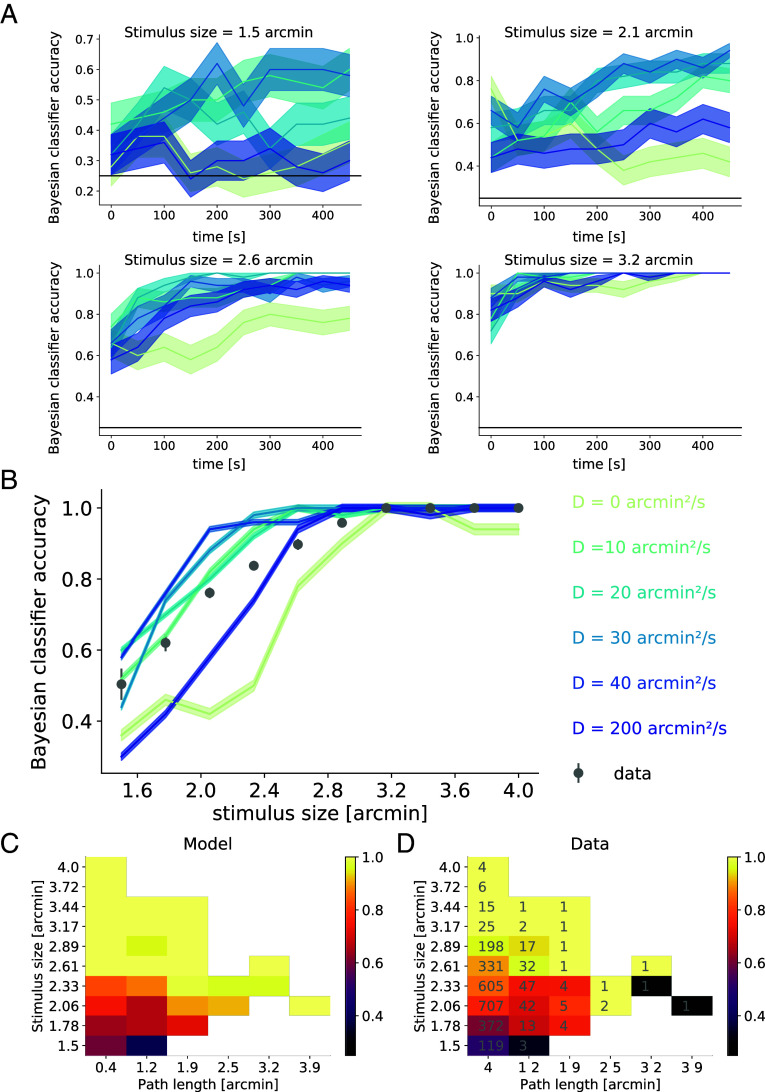
Bayesian classifier predicts that accuracy in visual discrimination tasks nontrivially depends on stimulus size and FEM amplitude, which is verified in empirical data on a trial-by-trial basis. (*A*) Classifier accuracy as a function of time for different values of D (darker blue: larger D) and different stimulus sizes (decreasing size from top to bottom and left to right). The horizontal black line denotes the chance-level fraction correct at 0.25. All fractions of correct discrimination increase over time. (*B*) Empirical (gray circles) and model-predicted (blue lines, darker blue for larger D) fraction of correct discrimination after 500 ms of simulation, as a function of stimulus size. The fraction of correct discrimination increases with stimulus size within a similar range for data and model, however vanishing FEM ($D=0\;{\mathrm{arcmin}}^{2}/s$) as well as larger-than-empirical-amplitude FEM ($D=200\;{\mathrm{arcmin}}^{2}/s$) impair accuracy across stimuli. (*C* and *D*) Heatmap of fraction of correct discrimination in bins of path length and stimulus size in the model (*C*) and data (*D*). For empirical values, the numbers in gray denote the number of trials per bin. Only bins where empirical path lengths were empirically recorded for those particular stimuli were represented. In these bins, the model predictions agree with empirical findings.

We found that evidence accumulation by the classifier from simulated spikes occurs for a large range of values of D. Indeed the fraction of correct discrimination increased over time, albeit at different rates depending on D and stimulus size (Fig. 3). For large stimuli (Fig. 3*A*), all values of D allowed for near-perfect accuracy, quantified by a fraction of correct discrimination nearing 1, except for $D=0\;{\mathrm{arcmin}}^{2}/s$; in this case, evidence accumulation was slow because cells spiked sparsely. Intermediate values of D yielded enhanced fractions of correct discrimination. Conversely, appreciably larger values of D resulted in impaired acuity, as both the accuracy of the classifier grew more slowly with time during the trial and the fraction of correct discrimination overall was lower for stimulus features smaller than the receptive field size. This can be understood in terms of the interplay between mechanisms: very small D does not allow refreshing receptive fields or encoding through differences in spiking rate (Fig. 2*C*, *Top*), while very large D prevents staying around the same position for long enough to average over noise from spiking activity and hampers the tracking of stimulus position (Fig. 2*C*, *Bottom*). The optimal value of D was observed to vary nontrivially with stimulus size due to interactions between the opposing coding mechanisms. Indeed, too small eye movements did not enable time-sustained coding with stimulus-relevant time fluctuations in RGC spiking rate, and too large values of D were detrimental to coding as averaging over noise is impaired when stimuli move too fast with respect to the retina. Overall, the interaction between mechanisms gave rise to an optimal value of D, which can be tested in empirical data.

We compared the fraction of correct 500 ms trials from simulations and human subjects. First, we computed the mean fraction of correct discrimination over all subject eyes and trials, as a function of stimulus size. As expected, both the model and subjects discriminated large stimuli more accurately. Across values of D, the model displayed good agreement with the data; as simulated fractions of correct discrimination across values of D lie in the same range as empirical ones, between 60% and 100% correct answers (Fig. 3*B*). It is also worth noting that the absence of FEM ($D=0\;{\mathrm{arcmin}}^{2}/s$, lightest teal line in Fig. 3*B*) yielded poorer results, in agreement with empirical evidence that stabilizing stimuli on the retina impairs the ability to discriminate between stimuli (4, 5).

To investigate for which stimuli FEM are the most helpful, we examined the fraction of correct discrimination of the subjects as a function of both stimulus size and path length of FEM, defined as the total amplitude of the stimulus trajectory with respect to the eye in a trial. In order to compare with empirical observations, we considered the simulated fraction of correct discrimination for a range of values of D matching the range found in subjects (Fig. 1*E*). Then, we computed a weighted average of the fraction of correct discrimination across values of D, where weights correspond to how heavily represented each value of D is across subjects (Fig. 1*E*). Once again, we found agreement between data and model (Fig. 3 *C* and *D*). For stimulus features, i.e., bars and spacings of the E, larger than the typical size of RGC receptive fields at the preferred position of fixation on the retina (stimulus sizes of 2.5 arcmin or more), discrimination was near-perfect across path lengths. However, for finer stimuli, our results suggested that the fraction of correct discrimination improved for larger path length, down to very fine stimuli at the limit of human acuity (1.5 arcmin).

Which mechanism explains the beneficial effect of FEM for visual decoding and acuity? The more immediate answer, namely that FEM refresh the image on the retina and thereby enhance spiking, was found to be insufficient. To illustrate this point, we repeated our theoretical study using modified model RGC with a unimodal temporal filter, such that the model RGC responds to light intensity rather than temporal variations of it (w=0 in Eq. **4**). In this case, RGC response was not transient, and FEM were no longer necessary to elicit sustained RGC spiking. In this modified model, acuity at classifying small stimuli was observed to deteriorate as expected, which confirms the relevance of the transient nature of RGC spiking to reproduce experimental results. Still, however, intermediate-amplitude FEM boosted the fraction of correct discrimination for stimulus features as fine as or finer than the receptive field size, consistent with empirical data (*SI Appendix*, Fig. S2). Thus, the “refresh mechanism” alone was not responsible for the benefits of FEM; in addition, FEM allowed more receptive fields to be exposed to stimuli, and hence for more RGCs to take part in conveying stimulus-relevant information. Further, we studied the effects of heterogeneity in the receptive field grid, which was shown to favor FEM for visual acuity in the literature (10). As shown in *SI Appendix*, Fig. S3, we observed that overall acuity is further hampered by vanishing FEM, consistently with previous work, as well as by much larger-than-empirical FEM. Together, the results suggested that coding through changes in spiking rate is a distinct mechanism behind benefits of FEM, distinct from the role of FEM to refresh receptive field contents as well as to move stimulus details out of the gaps of a heterogeneous receptive field mosaic.

In sum, our results showed that the empirical and model amplitude of FEM influences visual coding and discrimination on a trial-by-trial basis. Especially for stimuli finer than receptive fields, for which coding through changes in neural activity upon eye motion is especially relevant, longer FEM trajectories were favored. The model quantitatively captured experimental results by taking this mechanistic element into account. Indeed, our model explained how fine detail vision deteriorates in the absence of FEM, consistent with the experimental literature (5). Additionally, simulations quantitatively reproduced variations of the fraction of correct discrimination as a function of stimulus size and path length of FEM. Note that among model parameters, only D was fitted from empirically recorded FEM trajectories, and no parameters were adjusted to reproduce subjects’ fractions of correct discrimination. Surprisingly, our observations also suggested that the path length of empirical FEM appears to vary depending on stimulus size—are these variations significant, and if so, do they influence retinal coding?

**Fig. 4.**
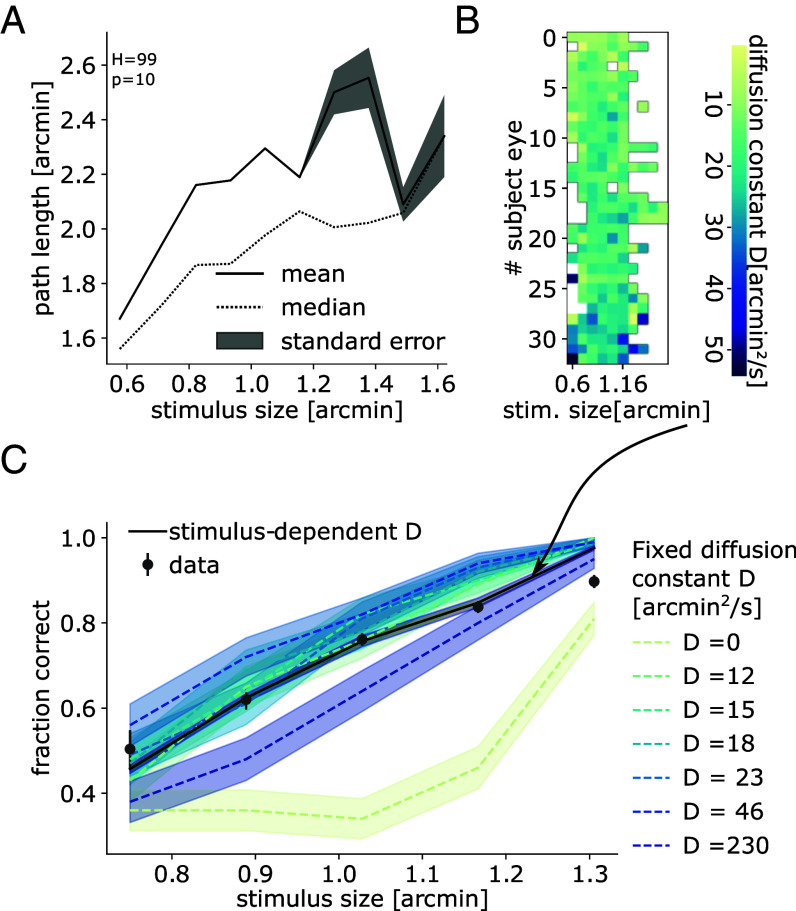
Variations in subjects’ FEM amplitude depending on stimuli remain within near-optimal range for retinal coding. (*A*) Path length statistics as a function of stimulus size: the mean (black line), SE of the mean (shaded area), and median (dotted line) are computed over all FEM trajectories for all subject eyes within bins of stimulus size. (*B*) Heat map of D values estimated for each subject eye and stimulus size, shown from subject’s eyes with the smallest mean D (*Top*) to those with the largest mean D (*Bottom*). (*C*) Model-predicted fraction of correct discrimination for empirical stimulus-dependent D (black solid line) and for a range of fixed values of D (dashed lines), alongside empirical acuity averaged across subject eyes as a function of stimulus size (gray dots). Lines represent means, and shaded areas represent SE in the mean.

In Fig. 4*A*, we observed that significant differences can be noted overall in path lengths across stimulus sizes (Kruskal–Wallis test, H=99, $P=10^{-17}$). In particular, statistical testing showed that path lengths for stimuli between 1.5 arcmin and 4 arcmin, near the subjects’ acuity threshold, were significantly different from others. To examine whether a modulation of the FEM occurs in single subjects, we have conducted an analogous analysis for each individual subject eye separately, in which we compared the distribution of lengths of FEM trajectory elements for different stimulus sizes (*SI Appendix*, Fig. S4). We found that in 36% of subject eyes, the amplitude of FEM varied significantly with stimulus sizes (Kruskal–Wallis test, P<0.05). Thus, while the modulation of FEM stimulus did not appear to occur systematically, it is observed in an appreciable fraction of the subjects.

In our experimental task, the delivery of stimuli was arranged in such a way that, in two successive trials, in a majority of cases (i.e., whenever the subject identified the correct stimulus orientation), the stimulus decreases in size. If FEMs were to decrease in amplitude over the duration of the experiment irrespective of stimulus identity (e.g., due to fatigue), our analysis of the correlation of FEM amplitude and stimulus size might have been affected by a confound. We verified that the modulation of FEM amplitude with stimulus size was not simply due to a simple monotonic decrease of the subjects’ FEM amplitude with time. Comparing consecutive trials in which the stimulus size increased (i.e., before and after a subject made a mistake, or before and after the beginning of a new trial block), we found no average decrease, but instead a significant increase in FEM amplitude as quantified by the end-to-end length of FEM trajectories (*SI Appendix*, Fig. S5). This analysis indicates that dependence of FEM amplitude on stimulus size was not due to a stimulus-independent suppression of the subjects’ FEM amplitude over the duration of the experiment. As subjects’ errors predominantly occur in response to small stimuli, the results further suggest that FEM amplitude tends to grow with stimulus size in the limit of small stimuli.

If FEM amplitude is consistently observed across subjects to vary with stimulus size for small stimuli, do these changes in FEM amplitude across stimuli help or harm retinal coding and visual acuity? Does changing FEM amplitude in a stimulus-dependent manner, as subjects do, benefit or impair visual acuity compared to keeping FEM amplitude coefficient at any given value?

Next, on a subject-by-subject level, we examined whether varying FEM amplitude across stimulus sizes, as subjects do, affects acuity, as compared to keeping FEM amplitude fixed. To that purpose (Fig. 4), we fitted D separately for each stimulus size and each subject (Fig. 4*B*). Using our model, ten trials were then simulated for each value of D per subject eye and per stimulus size, successively for empirical, averaged, and shuffled values of D. We compared this case to that of fixed D across stimulus sizes, with values chosen corresponding to the maximum, minimum, quartiles, and median of the empirical distribution as well as five times the maximum of the empirical distribution for comparison. For each value of D, we simulated as many trials as in the empirical-D simulations across subject’s eyes, i.e., 330 trials. From the fraction of correct discrimination (Fig. 4*C*), we observed that varying D across stimuli according to empirical values yielded an accuracy at the task similar to that of human subjects. Comparable acuity was also obtained with fixed D across stimulus sizes for all values of D save for the smallest and largest. For both these values, visual discrimination was found to be impaired relative to empirical accuracy and varying D as subjects do. Hence, the findings support that while FEM amplitude varies substantially, including with stimulus size, subjects tend to maintain their FEM amplitude within a range predicted by our model to be near-optimal for acuity.

One should note that free parameters in the model were not readjusted to resemble psychophysical results; as they were chosen to reflect RGC electrophysiological properties and kept constant throughout the manuscript. In particular, we observed that for a certain range of stimulus sizes smaller than foveal RGC receptive fields and near the limit of human acuity, the fraction of correct discrimination is enhanced on average by over a SE in the mean and up to 10% when modeling empirical, stimulus-dependent D compared to when modeling averaged, stimulus-independent D (Fig. 4*C*). For smaller stimulus sizes (1.5 arcmin or less), the subjects and model were found to perform poorly regardless of the values assigned to D. For stimulus bars and spacings larger than the typical receptive field size (stimulus sizes of 2.5 arcmin or more), the subjects and model performed near-perfectly across all values of D.

## Discussion

In this work, we investigated the conditions under which FEM help or harm retinal coding and visual acuity. Empirical FEM trajectories were recorded in healthy human subjects during a discrimination task, and empirical FEM amplitudes were estimated for all subjects and stimulus sizes (Fig. 1). FEM amplitude and its relation to stimulus size nontrivially affect acuity, through an intricate interplay of different mechanisms: averaging over spiking RGC activity, and temporal coding through refreshing the content of receptive fields, resulting in stimulus-informative variations in spiking (Fig. 2). Using the output of a model of retinal response to diffusing visual stimuli, an ideal Bayesian classifier successfully accounted for the benefits of FEM to discriminate fine stimuli (Fig. 3). The model of fraction of correct discrimination was found to qualitatively match human subject performance at the task, even though no model parameters were fitted to empirical fractions of correct discrimination, and only the diffusion coefficient of modeled FEM was fitted to recorded FEM trajectories. In addition, we noticed that subjects’ FEM path lengths varied with stimulus size. Our model showed that empirically observed variations in FEM amplitude remain within a range of values that yield near-optimal acuity, in that either smaller or larger FEM to those typically observed in subjects result in impaired acuity. The findings suggest that perception can benefit from being active even at the scale of FEM (Fig. 4).

While earlier modeling work on interpreting spikes in the presence of FEM had suggested stimulus encoding and decoding was possible in spite of FEM (2, 3), our work establishes that FEM are able to not only allow but also to enhance the encoding and decoding of task-relevant information, thereby accounting for experimental observations (5).

Furthermore, our work uncovers a relation between the (fine) spatial scale of visual stimuli and the role of the FEM amplitude. When one considers the opposing mechanisms of noise averaging (which favors lower values of D) and of the response dynamics (which favors higher values of D), nonzero, optimal value of D, varying with stimulus size, emerges. This notion of optimal FEM statistics for stimulus encoding is complementary with existing ideas that focused on stimulus statistics, suggesting that the spatial statistics of FEM, dominated by high frequencies, effectively transform the visual input so as to enhance our perception of fine detail (5, 12).

The results are in line with previous work (9) which demonstrated that artificial stimulus stabilization on the retina impaired visual acuity, while task performance was unaffected by manipulating stimulus trajectories as long as motion statistics remained similar to natural FEM. Our work also indicates that the knowledge of the detailed FEM trajectory is not needed to obtain enhanced acuity with as compared to without FEM of nonvanishing amplitude. Experimentally, our results suggest that their findings are also relevant for smaller stimuli in the 0.6 to 1.6 arcmin range. Computationally, our approach provides a complementary, model-driven understanding of the impact of FEM on visual acuity.

Our experimental findings also indicate that subjects can change the amplitude of their FEM during sustained fixation depending on stimulus size. This agrees with observations in the recent literature that empirical FEM statistics change in a task-dependent manner. In particular, the amplitude, speed, and curvature of FEM drift and microsaccades were reported to be distinct between passive viewing and acuity tasks (22). A high-acuity task using adaptive-optics-corrected stimuli also revealed that subjects adjusted drift length and direction to relocate stimuli closer to the topographical center of the foveal cone mosaic where sampling density is highest (11). In addition, recent work in primates supports that FEM originate from central neural circuitry generating noise that controls FEM statistics (23), which is consistent with subjects’ ability to modulate FEM amplitude according to stimuli. We also find that subjects vary their FEM amplitude while keeping it within a near-optimal range, but not at an optimal value, in line with previous literature emphasizing the suboptimality of human FEM statistics (24).

However, it is important to consider to what extent our experiments and model are relevant to natural viewing conditions. Stimulus presentation with the AOSLO compensates for optical aberrations of the eye that would otherwise blur out the stimuli on the retina. Hence, stimuli appear sharper in AOSLO experiments than images of the same size would appear in natural vision. To mimic natural viewing conditions, we have convolved the E letter stimuli with a kernel accounting for realistic blurring due to the optics of the human eye (25). For these naturalistic stimuli, our model outcomes are quantitatively different-evidence accumulation is slower and discrimination performance lower overall-but qualitatively similar (*SI Appendix*, Fig. S5). In particular, small-stimulus classification performance is still improved by intermediate amplitudes of FEM (*SI Appendix*, Fig. S5). It would be interesting to test model predictions (such as those summarized in *SI Appendix*, Fig. S5) in future AOSLO experiments with the use of artificially blurring out stimuli.

Moreover, our work has focused on a very simple stimulus set and task, where only four stimuli were considered: subjects knew that any stimulus was chosen from a set of four equiprobable ones, and the model made use of this highly constrained prior. By contrast, natural vision involves richer sets of possible visual stimuli and, thereby, incomparably broader prior distributions. (In many situations, still, priors are constrained by temporal continuity or context. For example, when reading we know that each letter is chosen from a known alphabet.) Natural vision might thus rely more heavily on evidence accumulation. It would be interesting to extend our experimental and theoretical framework to include broader sets of stimuli, to check the weighing of evidence vs. priors in human subjects. Though we find that changing FEM amplitude in a stimulus-dependent way, as subjects do, does not harm acuity, we do not uncover evidence that it helps beyond keeping FEM size coefficient at a given optimal value. Changing FEM size in a stimulus-dependent manner may support acuity in the context of more complex stimuli with features spanning spatial scales. This would also help relating our work to recent work relying on a similar Bayesian approach and making use of more complex stimuli and tasks, and focusing on the task of decoding natural images by estimating the probability that a given set of sparse features are present in them (10). This approach allowed us to identify additional mechanism through which FEM affect coding: namely, encoding can take advantage of spatial heterogeneity in the retinal receptive field mosaic through FEM. Our conclusions are complementary in that they exploit different mechanisms through which FEM enhance retinal coding.

While we have discussed coding mechanisms at the retinal level, other mechanisms and their associated costs may also incur, including motor costs in quelling noisy and jittery motion to reduce FEM amplitude. There may be conditions under which FEM amplitude is suboptimal, and acuity is enhanced by partially stabilizing stimuli on the retina (24). The perspective we propose on the effect of FEM on perception provides quantitative predictions for how subject acuity varies with FEM amplitude, to be tested in future psychophysical experiments in which FEM size may be effectively enhanced or reduced by amplifying or compensating for motion through eye tracking. More broadly, our findings suggest that active sensing is relevant even down to fine spatiotemporal scales where variability in eye motion and RGC spiking play a key role in shaping neural coding and sculpting human behavior.

## Materials and Methods

### Adaptive Optics Microstimulation.

High-resolution retina tracking during presentation of a small optotype was achieved by employing a custom AOSLO. In such a system, cone-resolved imaging and presentation of a visual stimulus is accomplished concurrently (26–28) (see *SI Appendix* for details).

### Visual Acuity Task and Retina Tracking.

All 17 adult participants (8 male, 9 female, age: 10 to 42 y) took part in a visual acuity task. Participants gave written informed consent before the experiments. All experimental procedures complied with the Declaration of Helsinki in its latest version and were approved by the independent Ethics Committee of the Rheinische Friedrich-Wilhelms-Universität of Bonn (Reference number: 2018-09). Participants had to indicate the orientation of a Snellen-E stimulus, presented randomly at one of four orientations (up, down, left, right). Each stimulus was presented for 500 ms at the center of the AOSLO raster after the participants initialized a trial with a keyboard press. Stimulus size during a trial was fixed and was changed between trials following a Bayesian adaptive staircase procedure [QUEST (29)]. Stimulus size decreased if the most recent response was correct and increased at incorrect responses. Each experimental run consisted of 20 trials yielding stimulus sizes roughly between 0.6 and 1.6 arcmin. More than 20 trials were conducted in case some trials had to be discarded due to eye blinks, saccades, or eye tracking artifacts. Experimental runs were repeated five times for each eye of each participant (except one participant in whom only one eye was tested). With each stimulus presentation, a 1 s AOSLO video was recorded, from which retinal motion and stimulus position were extracted (30, 31). Retinal motion and thus eye motion traces during stimulation were extracted by a real-time, strip-wise image registration and stabilization technique (32), with effective temporal sampling of ∼960 Hz. Trials containing defects of eye-tracking stabilization or microsaccades were identified by visual inspection (see *SI Appendix* for details).

### Retinal Response Model.

Modeled RGC receptive fields are arranged into a mosaic reminiscent of Fig. 1*A*, which we simulate as a square lattice with jittered center positions and receptive field sizes. Each receptive field is characterized by a spatial and temporal kernel. The spatial kernel is Gaussian with circular symmetry. The temporal kernel accounts for the fact that spiking activity decays to silence when the same fixed stimulus is shown within a time scale of the order of dt=50ms (2) and is implemented by subtracting the light intensity observed 50ms ago, weighted by a kernel weight, from the current intensity within the receptive field. Subsequently, to obtain RGC spiking rates, we multiply the stimulus after applying the spatiotemporal receptive field kernel by a constant gain, and add a baseline that controls RGC spiking rate in obscurity. Then, spikes are drawn from a Poisson process with said spiking rate. Eq. **4** describes RGC spiking rate and spiking probability in response to light stimuli.

[2] $$r(t)=r_{0}+\text{Δ}r(\int \int dxdyS(x_{t},y_{t})RF(x,y)$$

[3] $$-w\int \int dxdyS(x_{t-1},y_{t-1})RF(x,y)),$$

[4] $$\begin{array}{c} P\left(\sigma \left(t\right)=n\right)=\frac{r{\left(t\right)}^{n}}{n!}e^{-r(t)} \end{array}$$

with r(t) the RGC’s spiking rate at time t, $r_{0}=20$ Hz its baseline spiking rate in the absence of stimuli, Δr=120 Hz its gain controlling sensitivity to light intensity, RF its spatial receptive field specific to each neuron 0<w<1 the temporal kernel weight, $S(x_{t},y_{t})$ the two-dimensional stimulus centered onto position $\left(x_{t},y_{t}\right)$ the coordinates of the stimulus at time t, σ(t) the number of spikes fired by the RGC at time t, and n a nonnegative integer. For computation purposes, all possible positions (x,y) are described by coordinates upon a discrete square, grid and cyclic boundary conditions are applied. Parameter values for dt, $r_{0}$, and Δr are chosen based on ex vivo recordings from foveal RGCs (21). RGC firing rates were scaled due to the different light contrasts in our AOSLO experiment and in published experiments (21). This was based on in vivo recordings from neurons that receive direct input from RGCs and display firing-rate dependence on visual contrast (33, 34). These measurements suggest that firing rate scales linearly with contrast within the window of stimulus contrasts used in our experiments.

## Supplementary Material

Appendix 01 (PDF)

## Data Availability

Retinal imaging, motion tracking, and psychophysical data, as well as simulation codes are available on Mendeley Data (35).
