# Supplementary material for: Fixational eye movements as active sensation for high visual acuity
Source: Proc Natl Acad Sci U S A. 2025 Feb 4;122(6):e2416266122. doi: 10.1073/pnas.2416266122 (PMC11831129; doi:10.1073/pnas.2416266122)
Supplement: Supplementary file 1 — Appendix 01 (PDF) [file pnas.2416266122.sapp.pdf]

Supplementary Information for

Fixational eye movements as active sensation for high visual acuity

Trang-Anh E. Nghiem, Jenny L. Witten, Oscar Dufour, Wolf M. Harmening, Rava Azeredo da Silveira

Corresponding Authors: Trang-Anh E. Nghiem, Rava Azeredo da Silveira.
E-mail: trang-anh.nghiem@cantab.net, rava@ens.fr

This PDF file includes:

Supplementary text
Figs. S1 to S7 (not allowed for Brief Reports)
SI References

Supporting Information Text

Adaptive optics microstimulation. High resolution retina tracking during presentation of a small optotype was achieved by employing a custom adaptive optics scanning laser ophthalmoscope (AOSLO). In such a system, cone-resolved imaging and presentation of a visual stimulus is accomplished concurrently. The techniques have been described earlier (1–3), we mention only pertinent details here. In brief, the output of a supercontinuum light source (SuperK Extreme, NKT Photonics, Denmark) was spectrally filtered to create a red visible light channel, used for imaging, ocular wavefront sensing and microstimulation (center wavelength = 788 ±12 nm, FF01-788/12-25, Semrock, Rochester, USA). Adaptive optics correction, run in closed loop operation at about 25 Hz, consisted of a Shack-Hartmann wavefront sensor (SHSCam AR-S-150-GE, Optocraft GmbH, Erlangen, Germany) and a magnetic 97-actuator deformable mirror (DM97-08, ALPAO, Montbonnot-Saint-Martin, France). The imaging/stimulation beam was point-scanned across the retina, spanning a square field of 0.85 x 0.85 degrees of visual angle. The light reflected from the retina was detected in a photomultiplier tube (H7422-50, Hamamatsu Photonics, Hamamatsu, Japan) which was placed behind a confocal pinhole (pinhole diameter = 20 μm, equaling 0.5 Airy disk diameters). PMT signals were sampled at 20 MHz by a FPGA board (ML506, Xilinx, San Jose, USA), producing digital video frames at ~ 30 Hz with a spatial resolution of 600 pixels per degree of visual angle. By modulating the intensity of the imaging beam by an acousto-optic modulator (TEM-250-50-10-2FP, Brimrose, Maryland, USA), visual stimuli were created (thus a ‘light off’ stimulus within the visible scanning background, see main paper Fig. 1a).

Data pre-processing. Trials containing defects of eye-tracking stabilisation or microsaccades were identified by visual inspection of stabilized AOSLO videos and eye motion trajectories obtained from eye-tracking. Trials presenting sheared or trapezoid video frames associated with trajectories displaying large displacements within a single time frame were identified as stabilization failures and were discarded from subsequent analyses. For analysis, eye motion trajectories were downsampled to a 50 ms time bin to average over the AOSLO scanning over the recording frame pixel per pixel over 33 ms cycles.

Diffusion process and end-to-end length. Let $\boldsymbol{R}$ be the vector connecting the initial position of the eye to its position after N_t steps of duration Δt. We can write $\boldsymbol{R} = \sum_{i=1}^{N_t} \boldsymbol{r_i}$, where the displacement vector at each of N_t steps is given by $\boldsymbol{r_i} = l(X_i \boldsymbol{u_i} + Y_i \boldsymbol{v_i})$; X and Y are iid Poisson random variables with mean a and variance equal to the mean, ℓ is the smallest possible non-zero step size, $\boldsymbol{u_i}$ is a vector of norm 1 and phase drawn equiprobably from $\{0, \pi\}$ and respectively $\boldsymbol{v_i}$ is a vector of norm 1 and phase drawn equiprobably from $\{-\pi/2, \pi/2\}$. We compute the variance of the component of $\boldsymbol{R}$ along the x axis, $R_x = \sum_{i=1}^{N_t} \ell X_i u_{i\,x}$.

$$\mathrm{Var}(R_x) = \langle {R_x}^2 \rangle - \langle R_x \rangle^2 = \langle {R_x}^2 \rangle \tag{1}$$

by symmetry of $\boldsymbol{R_x}$ around 0 due to equiprobable leftward and rightward displacements.
The second moment can be calculated as follows:

$$\begin{aligned}
\langle {R_x}^2 \rangle &= \ell^2 \sum_i \sum_j \langle X_i X_j \rangle \langle u_{i\,x} \cdot u_{j\,x} \rangle & [2] \\
&= \ell^2 \left\langle \sum_i \sum_j X_i X_j u_{i\,x} \cdot u_{j\,x} \right\rangle & [3] \\
&= l^2 \sum_i \sum_j \langle X_i X_j \rangle \, \delta_{ij} & [4] \\
&= \ell^2 \sum_i \left\langle X_i^2 \right\rangle & [5] \\
&= N_t \ell^2 \langle X^2 \rangle & [6] \\
&= N_t \ell^2 [\langle X \rangle^2 + \mathrm{Var}(X)] & [7] \\
&= N_t \ell^2 (a^2 + a), & [8]
\end{aligned}$$

where we have used the properties of the Poisson process, X.

To compute a from $\langle {R_x}^2 \rangle$, we solve the quadratic equation and retain the positive solution, $a = \frac{\sqrt{1 + 4\frac{\langle {R_x}^2 \rangle}{N_t l^2}} - 1}{2}$. In addition, we recall that $a = 2D\Delta t$. Therefore, by fitting the slope α of the square end-to-end length as a function of time, one can express the diffusion coefficient as a function of α, as

$$D = \frac{\sqrt{1 + 4\frac{\alpha \Delta t}{\ell^2}} - 1}{4\Delta t}. \tag{9}$$

By symmetry, the same is true for the trajectory projection along the y axis.

Diffusion coefficient fitting. The diffusion coefficient can be estimated from the data by using Eq. 9, with $\ell = 0.1$ arcmin the minimum step size we used in discretizing FEM and α obtained by fitting the slope of the square end-to-end length in either x or y axis as a function of time. We note that if FEM were realizations of perfect random walks, with each step's direction uncorrelated from the previous one, the same D value would be obtained by fitting from either FEM trajectory path lengths or end-to-end lengths. However, small discrepancies in D values may arise. We approximate FEM as a random walk in the interest of maintaining reasonable computational burden for Bayesian classification, even though evidence suggests that small directional correlations exist at longer time scales than our 50 ms time step, rendering FEM trajectories slightly smoother than those described by our simple random walk model (4, 5).

Power-spectral density. We want to compute the power-spectral density, $S(f)$, of the random iid process, R_x (and equivalently R_y). By the central limit theorem, as N_t tends to infinity, R_x is well approximated by a Gaussian continuous random variable $W(t)$, of mean 0 and variance $\ell^2(a+a^2)\frac{t}{\Delta t}$ (Eq. 8, with $t = N_t \Delta t$). In the continuous limit, we have:

$$R_x = W(t) = \int_0^t \xi(s)\mathrm{d}s, \tag{10}$$

where $\xi(s)$ is continuous white noise whose moments are $\langle \xi(s) \rangle = 0$ and $\langle \xi(s)\xi(s') \rangle = \frac{l^2(a+a^2)}{\Delta t}\delta(s-s')$. The power-spectral density is obtained as (6, 7):

$$S(f) = \lim_{T\to+\infty} \left\langle \mid \widehat{W_T(t)} \mid^2 \right\rangle, \tag{11}$$

where $\widehat{\ }$ denotes the Fourier transform and $W_T(t)$ is $W(t)$ truncated at time T. Explicitly, $W_T(t) = W(t)\mathbb{1}_{[0,T]}(t)$ where $\mathbb{1}$ is the indicator function of the interval $[0, T]$. We use the following convention for the Fourier transform:

$$\widehat{W_T(t)}(f) = \frac{1}{\sqrt{T}} \int_0^T e^{-2i\pi f t} W(t)\mathrm{d}t. \tag{12}$$

$S(f)$ can then be written as

$$S(f) = \lim_{T\to+\infty} \frac{1}{T} \int_0^T \mathrm{d}t \int_0^T \mathrm{d}t' e^{2i\pi f(t-t')} \langle W(t)W(t') \rangle. \tag{13}$$

From Eq. 10, we can compute, the correlation term: $\langle W(t)W(t') \rangle = \int_0^t \int_0^{t'} \langle \xi(s)\xi(s') \rangle \mathrm{d}s\ \mathrm{d}s' = \frac{l^2(a+a^2)}{\Delta t}\min(t, t')$. To compute $S(f)$, first we divide the double integral on the square into an integral on the upper triangle and another on the lower triangle, to deal with the term $\min(t, t')$. Then, recognizing that one double integral is the conjugate of the other, we rewrite the sum as twice the real part. The double integral is then calculated as:

$$\begin{aligned}
S(f) &= \lim_{T\to+\infty} \frac{1}{T} \frac{l^2(a+a^2)}{\Delta t} \int_0^T \mathrm{d}t \int_0^T \mathrm{d}t' e^{2i\pi f(t-t')} \min(t, t') \\
&= \lim_{T\to+\infty} \frac{1}{T} \frac{l^2(a+a^2)}{\Delta t} \left[\int_0^T \mathrm{d}t \int_0^t \mathrm{d}t' e^{2i\pi f(t-t')} t' + \int_0^T \mathrm{d}t' \int_0^{t'} \mathrm{d}t\ e^{2i\pi f(t-t')} t \right] \\
&= \lim_{T\to+\infty} \frac{2}{T} \frac{l^2(a+a^2)}{\Delta t} \mathrm{Re} \left[\int_0^T \mathrm{d}t \int_0^t \mathrm{d}t' e^{2i\pi f(t-t')} t' \right] \\
&= \lim_{T\to+\infty} \frac{2}{T} \frac{l^2(a+a^2)}{\Delta t} \left[\frac{T}{(2\pi f)^2} - \frac{\sin(2\pi f T)}{(2\pi f)^3} \right] \\
&= \frac{2l^2(a+a^2)}{\Delta t (2\pi f)^2}.
\end{aligned} \tag{14}$$

The power spectrum is inversely proportional to the square of the frequency. By symmetry, the same is true for the trajectory projected along the y axis.

Retinal model parameter choice. Parameter values for r_0 and Δr were chosen based on the minimum and maximum spiking rates of foveal RGCs from multi-electrode array recordings of the *ex vivo* primate retina (8). As the visual stimulus contrast used by (8) was half of that used in our AOSLO experiment, we scaled our parameters based on reports of how the spiking rate varies with contrast in recordings of *in vivo* neurons which receive direct input from RGCs. The minimum spiking rate, r_0, does not vary with contrast, and the maximum spiking rate, on which Δr depends, scales linearly with stimulus contrast within the range of contrasts considered here (9, 10). For the stimulus contrast used in our experiments, we hence chose $r_0 = 20$ Hz and $\Delta r = 120$ Hz such that the maximum spiking rate is 140 Hz, i.e., double the maximum rate recorded in (8).

For the simulation time step, we chose $dt = 50$ ms, and as a result, we approximated the bimodal receptive field by a doubly rectangular receptive field with a positive and negative lobe. We made this approximation so that the computations at each time step of the Bayesian classification would remain tractable. Indeed, $N_x^T N_y^T$ operations need to be performed at every

time step to obtain the probability of spiking given past stimulus positions and update the posterior distribution, with T the number of time steps the temporal kernel spans over, so this calculation becomes computationally prohibitive for any value of T sensibly larger than 2.

Moreover, we are also limited in our choice of dt by experimental constraints. First, the diffusion coefficient, D, is extracted from empirical data or chosen to be comparable to empirical data, and the time taken by the AOSLO to scan the retina within a spatial window, which is used to measure FEM trajectories and thereby estimate D, is 30 ms. Second, the time scale at which autocorrelations in FEM-trajectory angles decay is also of the same order as the time step we chose: the angle of one trajectory element is uncorrelated with the angle of the trajectory element at the next time step (Fig. S1). Decorrelation between consecutive trajectory element angles at that time scale allows us to model FEM as a diffusion process with independent jump directions and sizes in successive time steps.

Further, comparing simulated retinal responses with a simplified temporal kernel, with time step $dt = 50ms$, against responses with a smooth, high-resolution temporal kernel, with time step $dt = 0.1ms$ following the formula from (11) (Fig. S7a), we find that spike counts are qualitatively similar (Fig. SS7b-c) and significantly correlated across RGCs and time (Pearson test, $r = 0.3$,$^{***}p = 10^{-6}$). The results suggest that our simplified approximation for temporal kernel filtration appears to capture the spatial and temporal fluctuations of simulated dynamics with a higher-resolution temporal kernel.

Bayesian classifier. The classifier aims to output the stimulus orientation, λ, by accumulating evidence from RGC spiking over time. Initially, the prior distribution is flat, i.e., $P(\lambda, x, y) = \frac{1}{4N_x Ny}$ for each of the 4 possible stimulus orientations (top, bottom, left or right), each of the N_x possible positions along the x axis and each of the N_y possible positions along the y axis. Since the temporal kernel is non-vanishing over two time steps and the diffusion process is iid, the current position of the stimulus depends only on the position at the previous time step, and spikes from the current time step only carry information about stimulus position during the current and previous time step. The posterior distribution can therefore be updated from the following:

$$
\begin{aligned}
P(\lambda, x, y|\{\boldsymbol{\sigma}_{t'}\}_{t' \le t}) &= \frac{1}{P(\{\boldsymbol{\sigma}_{t'}\}_{t' \le t})} P(\lambda, x, y, \{\boldsymbol{\sigma}_{t'}\}_{t' \le t}) && [15] \\
&= \frac{1}{P(\{\boldsymbol{\sigma}_{t'}\}_{t' \le t})} \int \mathrm{d}x'\mathrm{d}y' P(\lambda, x, x', y, y', \{\boldsymbol{\sigma}_{t'}\}_{t' \le t-1}, \boldsymbol{\sigma}_t) && [16] \\
&= \frac{1}{P(\{\boldsymbol{\sigma}_{t'}\}_{t' \le t})} \int \mathrm{d}x'\mathrm{d}y' P(\sigma_t|\lambda, x, x', y, y', \{\boldsymbol{\sigma}_{t'}\}_{t' \le t-1}) P(\lambda, x, x', y, y', \{\boldsymbol{\sigma}_{t'}\}_{t' \le t-1}) && [17] \\
&= \frac{1}{P(\{\boldsymbol{\sigma}_{t'}\}_{t' \le t})} \int \mathrm{d}x'\mathrm{d}y' P(\sigma_t|\lambda, x, x', y, y') P(x, y|\lambda, x', y', \{\boldsymbol{\sigma}_{t'}\}_{t' \le t-1}) P(\lambda, x', y', \{\boldsymbol{\sigma}_{t'}\}_{t' \le t-1})) && [18] \\
&= \frac{P(\{\boldsymbol{\sigma}_{t'}\}_{t' \le t-1})}{P(\{\boldsymbol{\sigma}_{t'}\}_{t' \le t})} \int \mathrm{d}x'\mathrm{d}y' P(\sigma_t|\lambda, x, x', y, y') P(x, y|x', y') P(\lambda, x', y'|\{\boldsymbol{\sigma}_{t'}\}_{t' \le t-1}), && [19]
\end{aligned}
$$

hence Eq. 1 of the main text. The transition matrix , $P(x, y|x', y')$, containing the probabilities of all possible x', y' given the currently considered x, y, is written as

$$
\begin{aligned}
P(x, y|x', y') = &\frac{d^{\frac{|x-x'|}{l}} e^{-d}}{\frac{|x-x'|}{l}!} + \frac{d^{N_x + \frac{|x-x'|}{l}} e^{-d}}{\left(N_x + \frac{|x-x'|}{l}\right)!} + \frac{d^{N_x - \frac{|x-x'|}{l}} e^{-d}}{\left(N_x - \frac{|x-x'|}{l}\right)!} \\
&+ \frac{d^{\frac{|y-y'|}{l}} e^{-d}}{\frac{|y-y'|}{l}!} + \frac{d^{N_y + \frac{|x-x'|}{l}} e^{-d}}{\left(N_y + \frac{|x-x'|}{l}\right)!} + \frac{d^{N_y - \frac{|x-x'|}{l}} e^{-d}}{\left(N_y - \frac{|x-x'|}{l}\right)!}
\end{aligned} \quad [20]
$$

where $d = 2D\Delta t$. The first three terms of the sum account for displacements along the x axis, where the first term captures the contribution of direct jumps from x', y' to x, y, the second term of longer jumps around the grid through the left, and the third term of longer jumps around the grid through the right due to cyclic boundary conditions. Similarly, the last three terms describe contributions from displacements along the y axis, including directly, around the grid through the top and the bottom.

Supplementary discussion. Between the fact that our eyes move and the transient nature of most retinal cells' light responses, it is difficult to state which exists to benefit visual coding as a consequence of the other. Nevertheless, some ecological factors can be taken into account. For fast exploration of the environment, regardless of the properties of neurons, eye movements are needed to scan the visual world and direct actions. The primary eye motion category for exploration is the saccade. If motion were organized as a collection of saccades separated by intervals of perfectly static, noiseless fixation, then one could argue that either transient or sustained cells would best support vision depending on the properties of saccades and the duration of fixations.

However, it has recently been argued, on the basis of data and modeling, that FEM are an unavoidable consequence of noise inherent to neural attractor dynamics in the central nervous system (12). If so, FEM are present regardless of whether they support vision. It is then natural to ask how RGC response dynamics shape and can support information encoding in the presence of FEM.

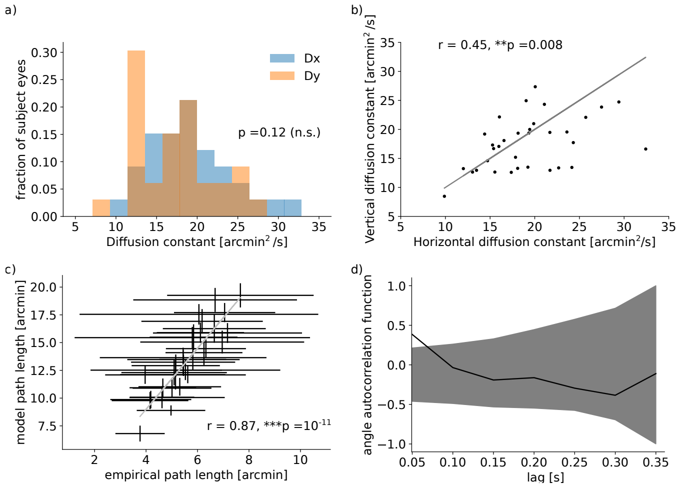

Fig. S1. Statistics of FEM trajectories across subjects. a) Diffusion coefficient along vertical axis, D_y, vs against horizontal axis, D_x. Each point is a subject eye. Significant correlations are found between D_x and D_y (Pearson test, p-value reported). b) Histogram of the distributions of D_x and D_y across subject eyes. No significant differences were identified between the two distributions (Wilcoxon test, p-value reported). c) Empirical path lengths per subject against model-generated path lengths with diffusion coefficient D fitted on FEM trajectories for that subject. Each point has coordinates the mean empirical and model-predicted for one subject, and error bars represent the empirical and model-predicted standard deviation across trials. For each subject, we simulated the same number of trials as was recorded experimentally. The grey line represents a linear fit (Pearson correlation and p-value reported). d) Autocorrelation function of the FEM trajectory angle with the horizontal axis, computed over all subject eyes and trials. Shaded areas correspond to one standard deviation from the mean over time-shuffled autocorrelation functions. Shuffling was performed across time bins separately in each trial, and mean and standard deviation over time-shuffled correlations for each time lag were computed over all shuffles of all trials for that time lag.

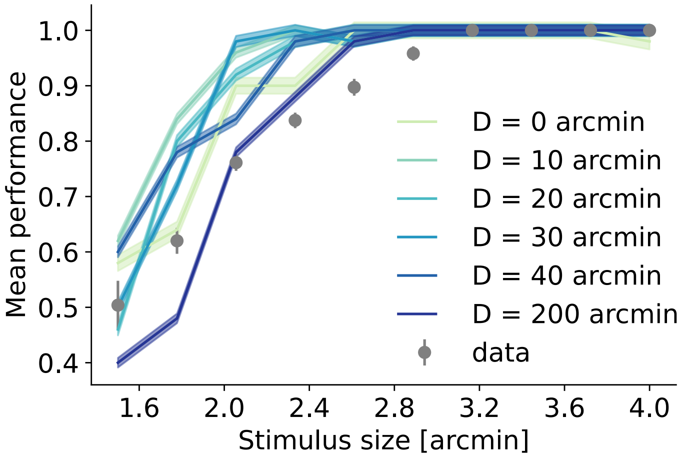

Fig. S2. Model and empirical fraction of correct trials for different FEM diffusion coefficient values as a function of stimulus size for sustained cells. Although acuity is improved for small to vanishing FEM compared to the case of transient cells, intermediate-length FEM trajectories still leads to improved fractions of correct trials compared to shorter trajectories.

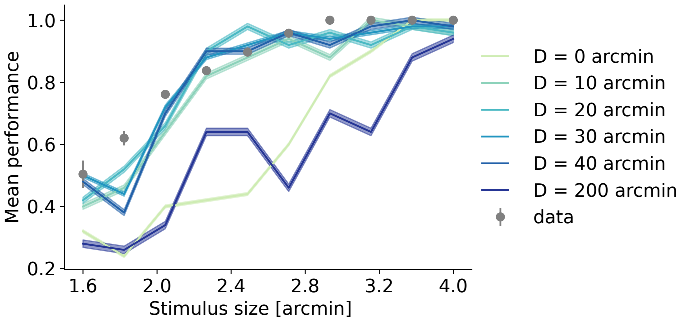

Fig. S3. Model and empirical fraction of correct trials for different FEM diffusion coefficient values as a function of stimulus size, with jittered receptive field positions. We note that compared to the non-jittered receptive field mosaic case, acuity is impaired for small to vanishing FEM amplitude, consistent with the literature, as well as very large FEM amplitude.

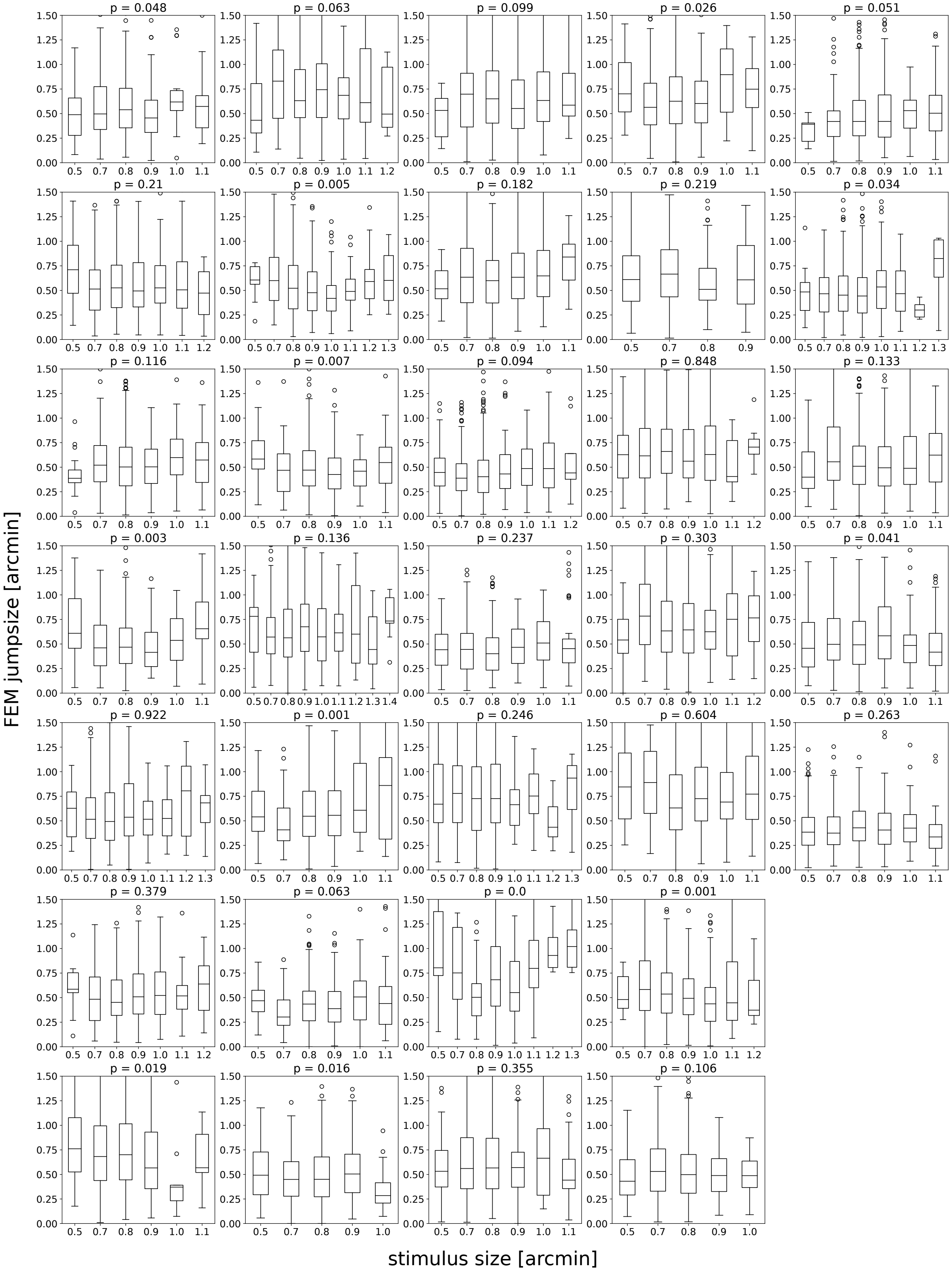

Fig. S4. Variation of the size of FEM element with stimulus size, for each subject eye. Each panel corresponds to a different subject eye. Trials are divided according to stimulus size, and for each stimulus size the distribution of lengths of FEM element (displacement of the eye due to FEM over 50 ms) is represented as a box plot. P-values are reported for a Kruskal-Wallis test evaluating whether the distributions vary significantly across stimulus sizes.

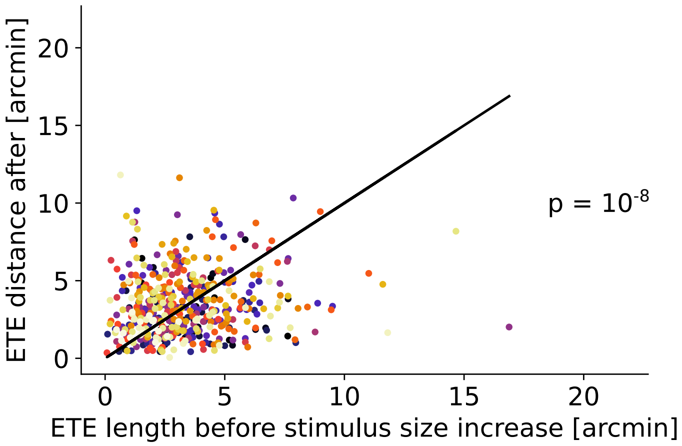

Fig. S5. End-to-end length of FEM trajectory after vs. before an increase in stimulus size from a trial to the next (for small stimuli after subjects made errors). Each point represents a pair of consecutive trials, each color represents a subject eye. The solid black line represents the identity. A significant increase of end-to-end length is observed when stimulus size increases between trials in 7 out of 33 subject eyes (Wilcoxon test, $^{*}p < 0.05$), as well as pooling all trials together across subjects (Wilcoxon test, $^{***}p = 10^{-8}$). None of the subject eyes exhibited a significant decrease in FEM trajectory end-to-end length between consecutive trials where stimulus size increased, which suggests that monotonic decrease of FEM amplitude through time cannot account for our empirical observations.

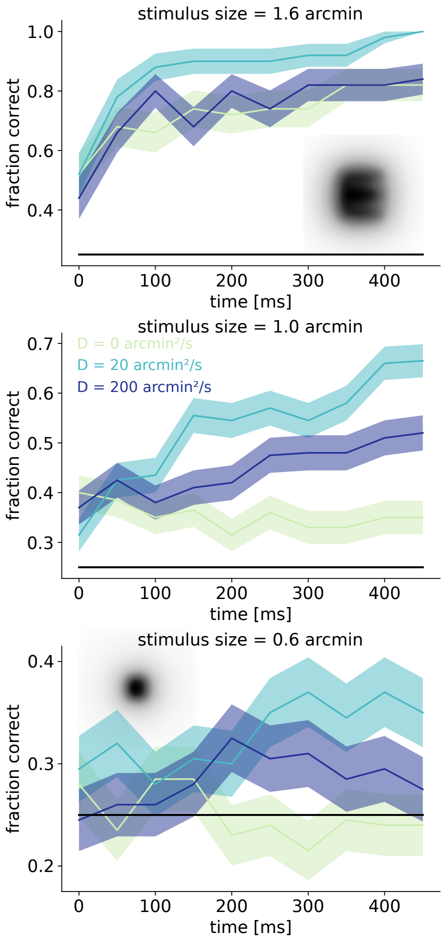

Fig. S6. Model accuracy at classifying stimuli blurred by the optics of the eye, for different stimulus sizes and FEM diffusion coefficients. The "E" letter stimuli are convolved with a kernel to simulate the way in which the optics of the eye blur out stimuli in natural viewing conditions (13). Different colors correspond to different values of the diffusion coefficient of the FEM, and each panel corresponds to a different stimulus size. Insets show stimuli of that size convolved with the optical blur kernel. These results show that the model accumulates evidence from blurred stimuli and, thereby, is accurate above chance. Intermediate values of the diffusion coefficient are most favorable to visual acuity in the presence of naturalistic blur.

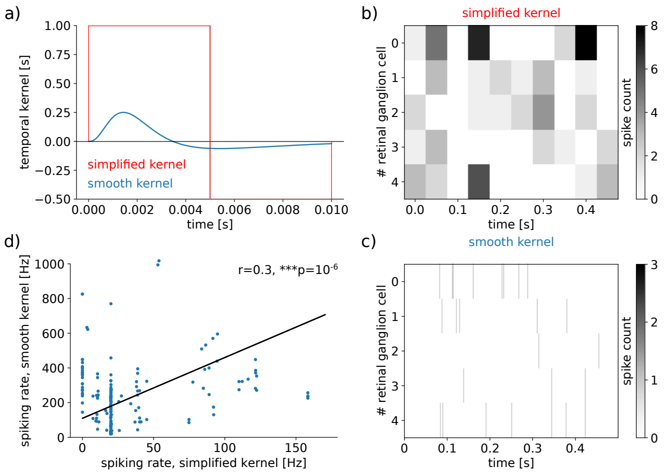

Fig. S7. Retinal response comparison for temporal kernel filtration at different time resolutions. a) Smooth (blue) and simplified (red) temporal kernels as a function of time. b-c) Raster plot of spike count against time for five example RGCs for simplified (b) and smooth (c) temporal kernel from the same simulated FEM trajectory, with $D = 20arcmin^2/s$ and stimulus size. d) Averaged spiking rate simulated with smooth temporal kernel over windows of 50 ms against spiking rate simulated with simplified kernel from the same simulated FEM trajectory. Each point represents one RGC in one time window. The solid line represents a linear fit (Pearson correlation and p-value reported).

References

1. S Poonja, S Patel, L Henry, A Roorda, Dynamic visual stimulus presentation in an adaptive optics scanning laser ophthalmoscope (2005).
2. A Roorda, et al., Adaptive optics scanning laser ophthalmoscopy. *Opt. express* **10**, 405–412 (2002).
3. WM Harmening, WS Tuten, A Roorda, LC Sincich, Mapping the perceptual grain of the human retina. *J. Neurosci.* **34**, 5667–5677 (2014).
4. MN Ağaoğlu, CK Sheehy, P Tiruveedhula, A Roorda, ST Chung, Suboptimal eye movements for seeing fine details. *J. vision* **18**, 8–8 (2018).
5. R Engbert, K Mergenthaler, P Sinn, A Pikovsky, An integrated model of fixational eye movements and microsaccades. *Proc. Natl. Acad. Sci.* **108**, E765–E770 (2011).
6. MP Norton, DG Karczub, *Fundamentals of Noise and Vibration Analysis for Engineers*. (Cambridge University Press, Cambridge), 2 edition, (2003).
7. D Krapf, et al., Power spectral density of a single Brownian trajectory: What one can and cannot learn from it. *New J. Phys.* **20**, 023029 (2018).
8. R Sinha, et al., Cellular and circuit mechanisms shaping the perceptual properties of the primate fovea. *Cell* **168**, 413–426 (2017).
9. DP Edwards, KP Purpura, E Kaplan, Contrast sensitivity and spatial frequency response of primate cortical neurons in and around the cytochrome oxidase blobs. *Vis. research* **35**, 1501–1523 (1995).
10. K Purpura, E Kaplan, R Shapley, Background light and the contrast gain of primate p and m retinal ganglion cells. *Proc. Natl. Acad. Sci.* **85**, 4534–4537 (1988).
11. Y Burak, U Rokni, M Meister, H Sompolinsky, Bayesian model of dynamic image stabilization in the visual system. *Proc. Natl. Acad. Sci.* **107**, 19525–19530 (2010).
12. N Ben-Shushan, N Shaham, M Joshua, Y Burak, Fixational drift is driven by diffusive dynamics in central neural circuitry. *Nat. Commun.* **13**, 1–13 (2022).
13. AB Watson, A formula for the mean human optical modulation transfer function as a function of pupil size. *J. Vis.* **13**, 18–18 (2013).
